# Absence of natural intracellular retinoids in mouse bone marrow cells and implications for PML-RARA transformation

**DOI:** 10.1038/bcj.2015.2

**Published:** 2015-02-27

**Authors:** H Niu, J Chacko, G Hadwiger, J S Welch

**Affiliations:** 1 Department of Internal Medicine, Washington University School of Medicine, St Louis, MO, USA

The X-RARA fusion proteins have reduced sensitivity to all-*trans*-retinoic acid (ATRA), and have been proposed to act by decreasing retinoid-dependent transcription required for myeloid maturation.^1, 2, 3^ We therefore sought to determine whether maturing myeloid cells are exposed to natural retinoid ligands *in vivo*. Surprisingly, we detected a paucity of natural retinoids capable of transactivating RARA-dependent transcription in adult mouse bone marrow cells, and the trace activity we observed tended to be in erythroid-lineage cells, rather than in myeloid-progenitors. This suggests that the resistance to retinoid-mediated transactivation likely has a limited role in X-RARA-dependent leukemogenesis because natural retinoids are largely absent during normal myeloid maturation.

The presence and distribution of natural retinoids have not been studied in adult hematopoiesis. To detect retinoids capable of transactivating RARA, we developed a UAS-GFP reporter mouse. UAS promoter sequences are recognized by the yeast Gal4 transcription factor, and are not activated by mammalian proteins (schema in Figure 1a). When the modular Gal4-DNA-binding domain is fused to the RARA ligand-binding domain, and expressed in UAS-GFP bone marrow cells, the reporter specifically detects intracellular retinoids that bind and transactivate RARA. This approach improves specificity of retinoid detection compared with alternative approaches, such as using the retinoid response element from the *RARB* promoter, which may respond nonspecifically to Rara/Rxra and Rarg/Rxra heterodimers, and Rxra/Rxra homodimers.

The mouse embryonic stem cell clone used to generate the UAS-GFP mice was selected through a series of functional assays, to determine responsiveness and background of the randomly integrated transgene (Supplementary Figure 1). We observed only trace background GFP expression in UAS-GFP mice in the absence of a Gal4 fusion protein, with the exception of a small population of GFP^dim^ lymphocytes in the peripheral blood and spleen (Supplementary Figure 2).

The UAS-GFP reporter was sensitive and specific to retinoids *ex vivo*. When Kit^+^ bone marrow cells were transduced with retrovirus expressing Gal4-RARA-IRES-mCherry (Gal4-RARA-IC) or Gal4-RARG-IRES-mCherry (Gal4-RARG-IC), we observed a dose-dependent response to *ex vivo* ATRA, with subnanomolar sensitivity (Figure 1a, Gal4-RARA-IC: EC_50_ 0.36±0.14 nm; Gal4-RARG-IC: EC_50_ 0.16±0.1 nm). This corresponds with the *in vitro*-measured K_d_ using radiolabeled ATRA (K_d_=0.2 and 0.2 nm, respectively).^4^ Synthetic, receptor-specific ligands induced receptor-specific GFP expression (Figure 1b, RARA-specific agonist: BMS753; RARG-specific agonist: BMS961). RARG binds to co-repressors with lower affinity than RARA.^5^ Consistent with this, Gal4-RARG-IC was modestly more sensitive to ATRA than Gal4-RARA-IC (Figure 1a), and Gal4-RARG-IC induced a small increase in the background GFP expression in the absence of exogenous ligand (Figure 1b column 5).

In order to determine whether bone marrow cells are exposed to natural retinoids *in vivo*, and whether this correlates with specific stages of hematopoietic differentiation, we transduced UAS-GFP Kit^+^ cells with Gal4-RARA-IC or Gal4-RARG-IC, and transplanted these cells into lethally irradiated recipient mice (the RARG vector was included to improve sensitivity of natural retinoid detection and to validate the RARA findings). The recipient mice were then maintained on standard chow (PicoLab 20, Labdiet, St. Louis, MO, USA, which contains 15 IU/g vitamin A) and then analyzed after complete engraftment (~6 weeks post-transplant). Surprisingly, we observed only trace GFP^dim^ bone marrow cells in mice transplanted with Gal4-RARA-IC, suggesting that few bone marrow cells have active intracellular retinoids, and these exist at or below the threshold of RARA transcriptional activation (Figures 1d and k). As a positive control, we treated mice with 50 or 200 μg ATRA in corn oil by gavage for 3 days (~7.5 and 30 mg/m^2^ per day), before analysis on day 4. We observed a dose-dependent induction in GFP expression, suggesting that most bone marrow cells can respond to active retinoids when they are present (Figure 1k).

Subgate analysis revealed that in the absence of exogenous ATRA, cells with trace retinoids (GFP^dim^ cells) correlated with erythroid-lineage cells (Ter119^+^CD71^+^), not with myeloid progenitors (Figure 2a). When mice were treated with 50 μg ATRA, retinoids were highest in myeloid progenitor (Kit^+^Gr1^+^ and Kit^+^CD11b^+^) and erythroid progenitor cells (Kit^+^CD71^+^ and Ter119^+^CD71^+^; Figure 2c). The largest difference between closely related populations was a 4.5-fold difference in the percent of GFP^+^ cells in progenitor cells (Lin^−^Kit^+^Sca^−^) vs Kit^+^Lin^−^Sca^+^ (KLS)-enriched stem cells (Lin^−^Kit^+^Sca^+^; *P*<0.005). In mice treated with 200 μg ATRA, the differences between specific progenitor compartments were blunted (1.7-fold difference between progenitor cells and KLS cells, *P*<0.001, Figure 2e).

We observed similar results in mice transplanted with Gal4-RARG-IC. We observed very limited evidence of natural retinoids in bulk bone marrow cells in the absence of exogenous ATRA (low percentages of GFP^dim^ cells; Figures 1h and k). Subgate analysis again found that the small population of cells with natural retinoids was biased toward populations of erythroid cells (Ter119^+^CD71^+^; Figure 2b). We again observed a difference in the percent of GFP^+^ cells in the progenitor cells vs KLS cells when the mice were untreated (14-fold, *P*<0.0001, Figure 2b), and this was diminished with 50 μg ATRA treatment (1.6-fold, *P*<0.1, Figure 2d), and abolished with 200 μg ATRA treatment (Figure 2f).

*RARA* is the target of at least 10 fusion proteins that lead to acute promyelocytic leukemia (APL),^6^ and *RARA* expression increases dramatically during myeloid maturation.^7^ Many of the *X-RARA* fusions have been proposed to act by reduced sensitivity to retinoid-dependent differentiation programs (a dominant negative effect).^1, 2, 3^ Therefore, we were surprised that the natural retinoids were largely absent during myeloid differentiation *in vivo* (Figures 2a and b). *Ex vivo,* UAS-GFP cells transduced with Gal4-RARA-IC or Gal4-RARG-IC responded to subnanomolar concentrations of ATRA, and *in vivo,* a short course of 50 μg ATRA was adequate to induce GFP response in all measured hematopoietic compartments, suggesting that the system should be adequately sensitive to detect physiologically relevant concentrations of intracellular natural retinoids *in vivo* (Figures 1a, 2c and f).

Aldehyde dehydrogenase (ALDH) expression and activity have been correlated with hematopoietic stem cells, and ALDH is the rate limiting step in ATRA synthesis.^8, 9, 10^ Observations that ATRA can augment stem cell function *ex vivo*^11, 12^ suggest that the stem cell-associated function of ALDH might occur through ATRA synthesis. However, our data suggest that KLS cells exist within a retinoid deplete environment, with disproportionately low intracellular retinoids, even when mice were treated with exogenous ATRA (Figures 2b and d). *In vivo* this likely occurs through stromal cell P450 activity, which is capable of rapidly eliminating local retinoids.^13^ Therefore, the stem-associated function of ALDH is not likely to be through ATRA generation.

We observed that even 200 μg of ATRA (given for short courses) was inadequate to activate retinoid-dependent transcription in all bone marrow cells in any hematopoietic compartment that we measured, and many cells remained mCherry^+^GFP^−^ (Figures 1f and j). Successful treatment of APL requires month-long courses of ATRA.^14^ Our data support this idea and suggest that long courses at pharmacologic doses may be necessary to efficiently activate retinoid-induced maturation within all leukemia cells. This may be especially important to adequately treat cells that reside in retinoid-deplete stromal cell niches (for example, leukemia stem cells).

The UAS-GFP reporter system has the advantage of being highly modular (which allows for the analysis of multiple nuclear receptor ligand-binding domains), but this requires retroviral expression of the Gal4-fusion protein and subsequent transplantation/engraftment. We are currently generating a Gal4-RARA transgenic mouse to circumvent this issue. In addition, because GFP induction by the Gal4-RARA fusion protein requires functional co-activators, the GFP read-out integrates both the intracellular concentration of active ligands, and the intracellular co-activator/co-repressor environment. Cell populations that lack appropriate co-activators to induce response to ligand, or that contain trans-repressive effects from other transcription factors, will exhibit diminished or absent response to ligands. Thus, the read-out integrates both local retinoid concentrations and the transcriptional sensitivity to retinoids of the intracellular environment.

This study has focused on intracellular retinoids that are present in bone marrow cells under steady-state conditions *in vivo*. It is unknown whether the expression of *PML-RARA* or other hematopoietic stimuli might alter intracellular retinoid production, and whether this might lead to a pan-bone marrow endocrine effect or to a lineage-restricted retinoid production. The erythroid progenitor bias observed (Figures 2b and d) at low doses of exogenous ATRA suggests that the erythroid-specific stimulation might lead to augmented intracellular retinoids, although this is largely speculative. Future studies will need to screen diverse hematopoietic stimuli to determine whether pathologic or physiologic processes induce *in vivo* retinoid availability in hematopoietic cells.

In conclusion, although X-RARA fusion proteins have been suggested to act by blocking retinoid-dependent transcriptional programs required for myeloid maturation, we observed a surprising paucity of natural retinoids capable of transactivating Gal4-RARA in primary mouse bone marrow cells *in vivo.* This suggests that these leukemic fusion proteins act predominantly through alternative mechanisms, and that retinoid-dependent transactivation likely has a limited role in normal, adult myeloid maturation.

## Figures and Tables

**Figure 1:**
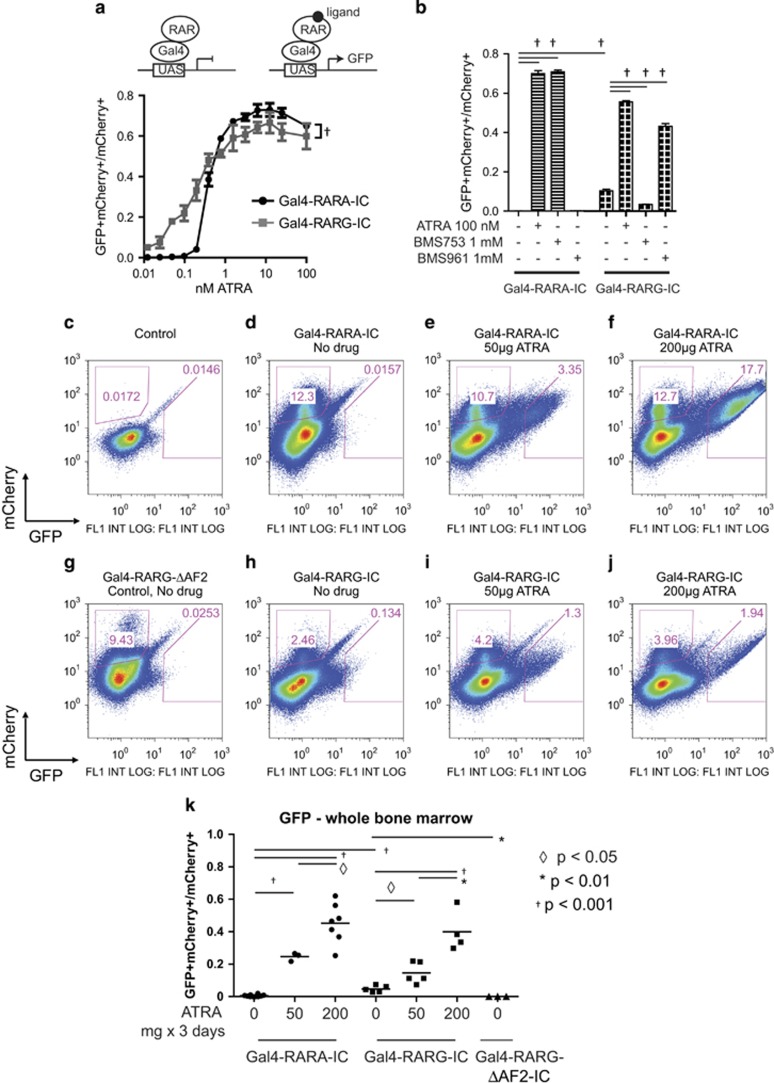
(**a**) Kit^+^ UAS-GFP bone marrow cells were selected via the Automacs Pro (Miltenyl Biotec, San Diego, CA, USA) and transduced with Gal4-RARA-IRES mCherry (Gal4-RARA-IC) or Gal4-RARG-IRES mCherry (Gal4-RARG-IC) retrovirus and treated with increasing concentrations of ATRA for 72 h in progenitor expansion medium (RPMI1640 medium, 15% fetal bovine serum, stem cell factor (50 ng/ml), interleukin-3 (10 ng/ml), Fms-related tyrosine kinase 3 ligand (25 ng/ml), thrombopoietin (10 ng/ml), l-glutamine (2 mM), sodium pyruvate (1 mM), HEPES buffer (10 mM), penicillin/streptomycin (100 units/ml) and β-mercaptoethanol (50 μm)). Retrovirus was produced as previously described.^15^ Statistical comparison performed via two-way anlaysis of variance. Schema describes the UAS-GFP reporter system. In the absence of retinoids, the fusion protein containing the Gal4-DNA-binding domain and a retinoid receptor ligand-binding domain binds to the UAS-GFP transgene, but does not induce GFP expression. In the presence of a retinoid ligand, the Gal4-fusion protein induces GFP expression. (**b**) Kit^+^ UAS-GFP bone marrow cells were transduced with Gal4-RARA-IC or Gal4-RARG-IC retrovirus, and treated with ATRA, BMS753 (an RARA-specific ligand that does not transactivate RARG) or BMS961 (an RARG-specific ligand that does not transactivate RARA). Cells were cultured in progenitor expansion medium for 72 h before analysis by flow cytometry. (**c**–**j**) Total bone marrow cells from either control UAS-GFP mice, or mice transplanted with Kit^+^ UAS-GFP bone marrow cells transduced with Gal4-RARA-IC, Gal4-RARG-IC or Gal4-RARG-ΔAF2-IC retrovirus (the ΔAF2 mutation removes helix 12 and prevents retinoid-dependent transactivation of GFP, acting as a negative control). Analysis was performed following engraftment. Mice treated with indicated doses of ATRA received daily ATRA in corn oil by gavage for 3 days and were analyzed on day 4. Cells along the mCherry/GFP median were excluded from mCherry^+^ and GFP^+^ gates, and correlated with autofluorescent granulocytes in control samples. (**k**) Summary of GFP^+^ cells observed within the bulk bone marrow cells. Each circle (transplanted with Gal4-RARA-IC retrovirus), square (transplanted with Gal4-RARG-IC retrovirus) or triangle (transplanted with Gal4-RARG-ΔAF2-IC retrovirus) represents the results from a separate mouse. In all the studies, the percentage of GFP^+^ cells was normalized within the total of mCherry^+^ and GFP^+^ cells (the cells that did respond vs the cells that potentially could respond). Flow cytometry analysis was performed with the FlowJo version 9 (FlowJo, LLC, Ashland, OR, USA) and statistical analysis was performed with the Prism (Graphpad, San Diego, CA, USA).

**Figure 2:**
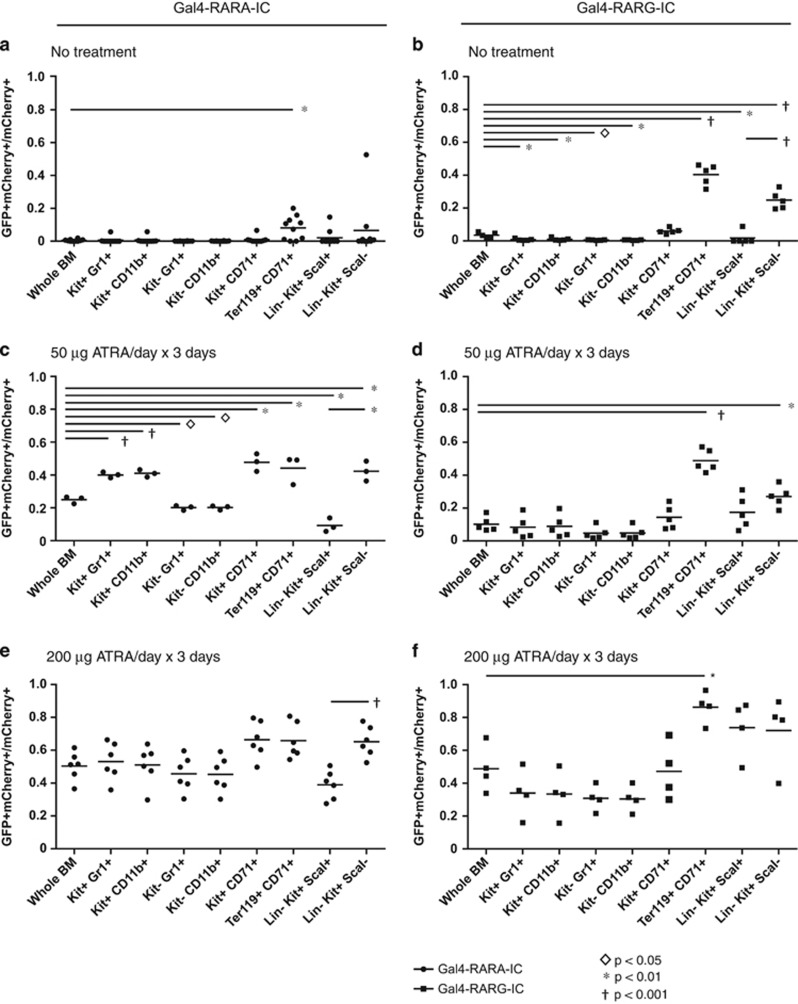
Hematopoietic distribution of natural retinoids that transactivate either Gal4-RARA or Gal4-RARG. Bone marrow cells from Figure 1k were stained as indicated for subgate analysis of hematopoietic populations. (**a**, **c** and **e**) Mice transplanted with Gal4-RARA-IC retrovirus. (**b**, **d** and **f**) Mice transplanted with Gal4-RARG-IC retrovirus. Each circle or square represents the results from a separate mouse.
