# Supplementary figures and images for: Absence of natural intracellular retinoids in mouse bone marrow cells and implications for PML-RARA transformation
Source: Blood Cancer J. 2015 Feb 27;5(2):e284–. doi: 10.1038/bcj.2015.2 (PMC4349261; doi:10.1038/bcj.2015.2)

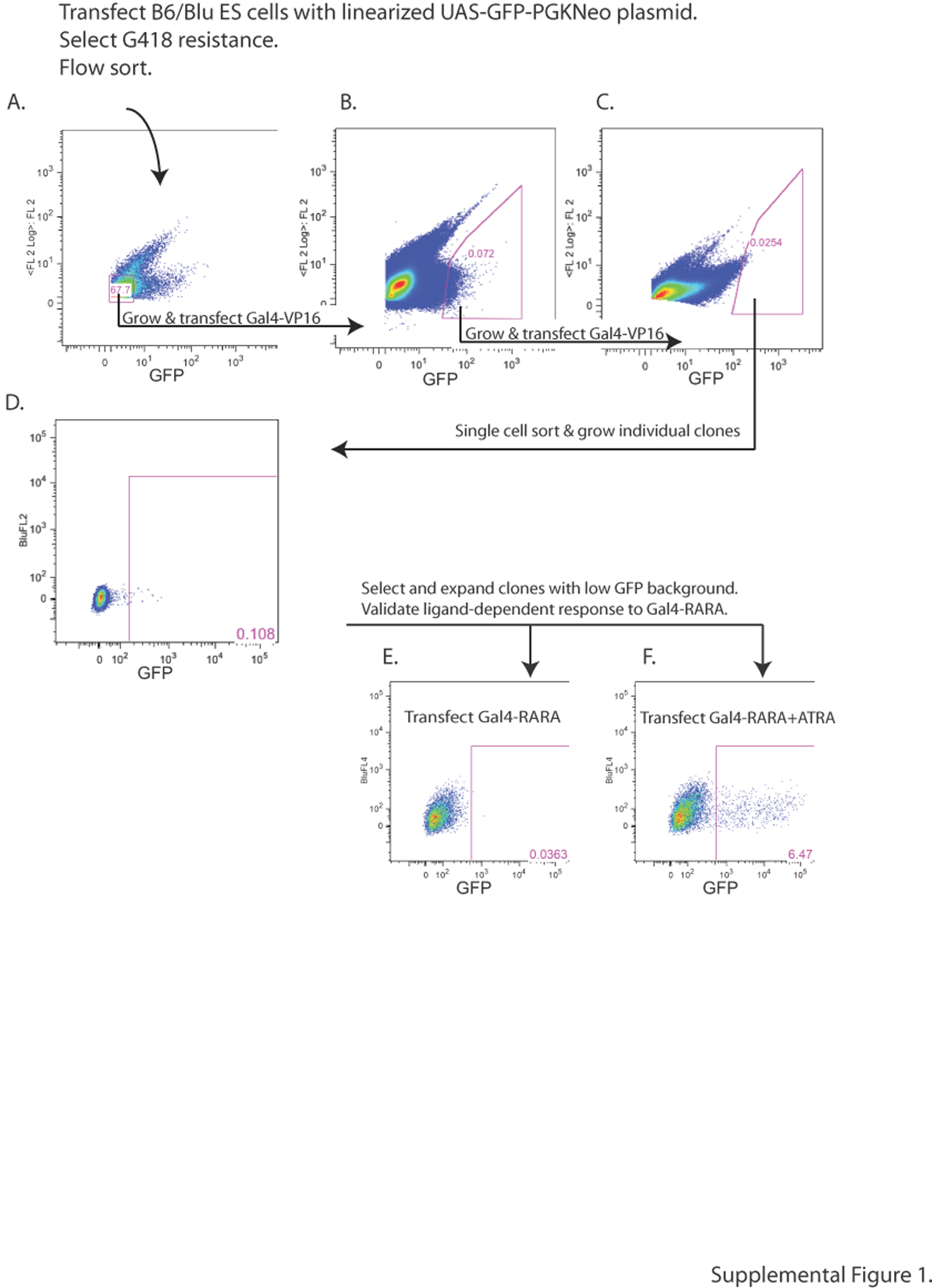

Supplement: Supplementary Figure 1 [file bcj20152x1.tif]

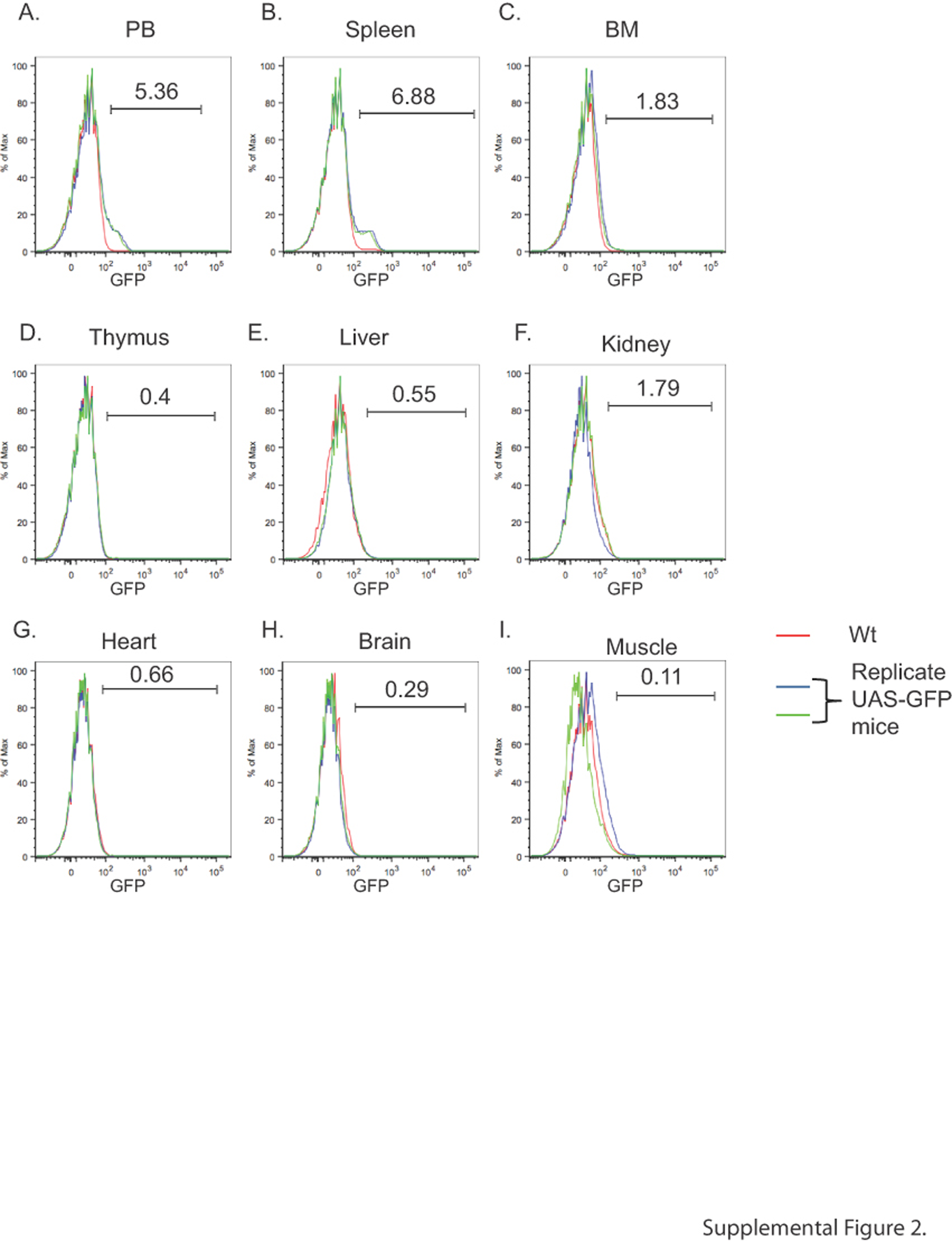

Supplement: Supplementary Figure 2 [file bcj20152x2.tif]
