# Supplementary material for: Absence of natural intracellular retinoids in mouse bone marrow cells and implications for PML-RARA transformation
Source: Blood Cancer J. 2015 Feb 27;5(2):e284–. doi: 10.1038/bcj.2015.2 (PMC4349261; doi:10.1038/bcj.2015.2)
Supplement: Supplementary Figure legends [file bcj20152x3.doc]

**Supplemental Figure 1.** Generation of UAS-GFP reporter mice. A. The UAS-GFP transgene (UAS-GFP-PGKNeo) was developed by cloning the BamHI-NotI eGFP cassette from eGFP-N1 (Clontech, Mountain View, CA) into the BamHI-NotI sites in pGene/V5-His B (Life Technologies, Grand Island, NY) (the V5-His tags are downstream of the eGFP-N1 stop site). A LoxP-PGK-Neo-LoxP cassette replaced the pGene/V5-HisB Zeocin DraIII cassette. The UAS-GFP-Neo plasmid was linearized and electroporated into B6/Blu ES cells. B6/Blu ES cells were then selected for G418 resistance, and flow sorted for GFP low expression. B. These cells were expanded, transiently transfected with Gal4-VP16 plasmid, and GFP^+^ cells sorted. C. These cells were expanded and again transiently transfected with Gal4-VP16 plasmid, and individual GFP^high^ cells sorted into 96 well plates. D. Individual clones were selected with low GFP expression in the absence of transfected Gal4-fusion plasmids. E. and F. Clones were transiently transfected with Gal4-RARA plasmid and treated without (E) or with (F) ATRA to identify clones with low background and appropriate response to Gal4-RARA. The low percent GFP^+^ cells is due to inefficient lipid-based transfection of ES cells.

**Supplemental Figure 2.**  Basal GFP expression in UAS-GFP mice. Indicated tissues from healthy wild type or UAS-GFP mice were harvested, single-cell suspensions generated, and analyzed by flow cytometry. Peripheral blood, bone marrow, spleen, and thymus cells were collected by flushing bones and crushing spleen and thymus tissues through a strainer. Kidney, liver, heart, brain and skeletal muscle were cut into 1-3 mm slices, digested in 0.1% collagenase IV at 37°C for 45 minutes.
